# EAAUnet-ILT: A Lightweight and Iterative Mask Optimization Resolution with SRAF Constraint Scheme

**DOI:** 10.3390/mi16101162

**Published:** 2025-10-14

**Authors:** Ke Wang, Kun Ren

**Affiliations:** 1 College of Integrated Circuits, Zhejiang University, Hangzhou 310058, China; 2 Zhejiang ICsprout Semiconductor Co., Ltd., Hangzhou 311200, China

**Keywords:** resolution enhancement technique, inverse lithography technology, lightweight model, mask constraint scheme

## Abstract

With the continuous scaling-down of integrated circuit feature sizes, inverse lithography technology (ILT), as the most groundbreaking resolution enhancement technique (RET), has become crucial in advanced semiconductor manufacturing. By directly optimizing mask patterns through inverse computation rather than rule-based local corrections, ILT can more accurately approximate target design patterns while extending the process window. However, current mainstream ILT approaches—whether machine learning-based or gradient descent-based—all face the challenge of balancing mask optimization quality and computational time. Moreover, ILT often faces a trade-off between imaging fidelity and manufacturability; fidelity-prioritized optimization leads to explosive growth in mask complexity, whereas manufacturability constraints require compromising fidelity. To address these challenges, we propose an iterative deep learning-based ILT framework incorporating a lightweight model, ghost and adaptive attention U-net (EAAUnet) to accelerate runtime and reduce computational overhead while progressively improving mask quality through multiple iterations based on the pre-trained network model. Compared to recent state-of-the-art (SOTA) ILT solutions, our approach achieves up to a 39% improvement in mask quality metrics. Additionally, we introduce a mask constraint scheme to regulate complex SRAF (sub-resolution assist feature) patterns on the mask, effectively reducing manufacturing complexity.

## 1. Introduction

**Figure 1:**
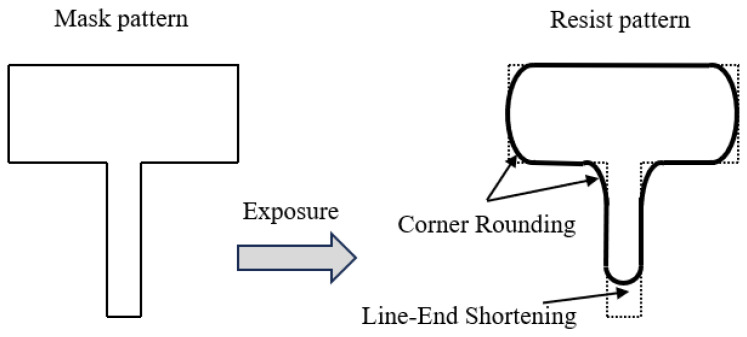
Line-end shortening and corner rounding resulted by optical proximity effect.

Photolithography, the most complex and costly process in semiconductor manufacturing, refers to the transfer of mask patterns onto photoresist through exposure to specific-wavelength light [1]. The resulting photoresist patterns ultimately define the circuit features etched on wafers, making lithography a pivotal step in advanced semiconductor fabrication. With the relentless progression of Moore’s Law [2], the continual scaling-down of critical dimensions has exacerbated diffraction and interference effects during lithography. These phenomena cause significant deviations between the printed patterns on silicon wafers and the original design [3], manifesting as line-end shortening, corner rounding (where right-angle structures become rounded) [4], and other distortions, as illustrated in Figure 1. Such effects are collectively termed optical proximity effects (OPEs).

To mitigate optical proximity effects (OPEs) in photolithography, optical proximity correction (OPC) techniques have been developed as a compensation strategy during mask design. Mainstream OPC approaches fall into two categories: (1) rule-based OPC [5] and (2) model-based OPC [6,7,8,9]. Rule-based OPC modifies mask patterns using pre-defined empirical rules derived from process test structures and historical data, such as adding serifs to isolated lines or applying edge bias to dense patterns. While deterministic and simulation-free, its reliance on extensive rule libraries becomes a bottleneck for complex masks, especially at advanced nodes. Model-based OPC employs lithography simulation models (e.g., optical and resist models) to predict wafer imaging results and iteratively adjusts mask patterns until simulations match target designs. By leveraging numerical optimization (e.g., gradient descent), it achieves higher accuracy than rule-based OPC for advanced nodes but requires substantial experimental data and computational resources for model calibration, often introducing modeling errors.

In contrast to conventional model-based OPC, inverse lithography technology (ILT) offers superior advantages. Model-based OPC follows a forward optimization approach, segmenting masks into edges for localized corrections, which inherently limits its solution space. ILT, however, inversely computes the optimal mask directly from the target pattern [10], enabling pixel-level optimization with higher fidelity and greater flexibility in generating sub-resolution assist features (SRAFs). This expands the process window and makes ILT particularly effective for high-pattern-repetition devices like DRAM and SRAM, reducing edge placement errors (EPEs), minimizing line-end shortening, and enhancing overall manufacturability.

The evolution of ILT implementations has witnessed a shift in research priorities. Early ILT development primarily addressed fundamental challenges including imaging accuracy, mask manufacturability, SRAF control, and process window stability. However, as ILT methodologies matured, computational efficiency emerged as the predominant bottleneck. Current ILT implementations primarily include gradient descent-based and deep-learning-based methods. In gradient descent ILT, Gao et al. [11] derived gradients for the L2 error between resist and mask images and the process variation band (PVB), formulating edge placement error (EPE) violations as sigmoid functions with closed-form gradients. Sun et al. [12] proposed a multi-resolution gradient descent to accelerate convergence, while Chen et al. [13] reduced simulation time via downsampled grids and sparse Fourier transforms, combined with Nesterov-accelerated gradients. However, gradient descent methods risk local minima convergence and remain computationally demanding.

Deep-learning-based ILT has advanced significantly to address these computational challenges. Ye et al. [14] introduced LithoGAN, an end-to-end GAN framework mapping masks to resist patterns. Jiang et al. [15] proposed Neural-ILT using a U-Net backbone with custom layers for iterative refinement. Zhu et al. [16] enhanced this via L2O-ILT, stacking adaptive custom layers for multi-objective optimization, significantly speeding up convergence. Despite these advances, the computational intensity of deep learning approaches remains a critical limitation.

Trade-offs: Gradient descent ILT relies on physical models without training data but suffers from local minima and slow convergence. Deep-learning ILT is faster but computationally intensive, and its physics-agnostic models may yield suboptimal masks with poor manufacturability. The pursuit of lightweight architectures has therefore become essential for practical ILT implementation.

Our solution: We present an ILT framework combining a lightweight EAAUnet (enhanced from AAUnet [17]) with iterative custom-layer refinement and SRAF constraints. EAAUnet integrates multi-scale convolutions and dual-attention mechanisms (channel + spatial) for efficient feature extraction, while regularization terms in custom layers improve optimization. Mask-filtering techniques enhance smoothness, and SRAF constraints balance complexity with imaging quality, specifically addressing the computational bottleneck while maintaining performance across traditional ILT challenge domains.

## 2. Preliminaries

### 2.1. Lithography Simulation Model

In the field of photolithography, Hopkins theory is widely employed for modeling the aerial image—defined as the light intensity distribution at the photoresist surface—within lithography simulation workflows. The imaging process is typically described by the Hopkins equation [18]:

(1) $$I\left(x,y\right)=\int {∭}_{-\infty }^{\infty }T\mathrm{CC}\left(f^{\prime },g^{\prime };f^{\prime \prime },g^{\prime \prime }\right)O\left(f^{\prime },g^{\prime }\right)O^{*}\left(f^{\prime \prime },g^{\prime \prime }\right)$$

$$e^{-i2\pi [\left(f^{\prime }-f^{\prime \prime }\right)x+\left(g^{\prime }-g^{\prime \prime }\right)y]}df^{\prime }dg^{\prime }df^{\prime \prime }dg^{\prime \prime }$$

where *O*(*f*′, *g*′) represents the Fourier transform of the mask pattern, ∗ denotes the complex conjugate operator, and TCC(*f*′, *g*′; *f*″, *g*″) is the transmission cross coefficient (TCC), which characterizes the joint transfer properties of the illumination source, mask, and projection lens system. The TCC is expressed as:

(2) $$T\mathrm{CC}\left(f^{\prime },g^{\prime };f^{\prime \prime },g^{\prime \prime }\right)=\iint_{-\infty }^{\infty }\tilde{J}(f,\;g)\tilde{H}\left(f+f^{\prime },g{\;+\;g}^{\prime }\right){\tilde{H}}^{*}\left(f+f^{\prime \prime },g{\;+\;g}^{\prime \prime }\right)df\;dg$$

where $\tilde{J}$(*f*, *g*) represents the intensity distribution of the partially coherent source, $\tilde{H}$(*f* + *f*′, *g* + *g*′) is the pupil function of the projection lens, and *f* and *g* denote spatial frequency coordinates. The TCC matrix is large-scale and exhibits significant sparsity, indicating room for further simplification. In practice, the Sum of Coherent Systems (SOCSs) method is widely adopted [19]. Based on singular value decomposition, it approximates the original matrix using a small number of eigenvalues and eigenvectors—effectively replacing a high-order system with a low-order model. As the TCC matrix is positive definite, it can be mathematically decomposed into a series of eigenvalues and corresponding eigenvectors, providing a theoretical basis for this approximation. By retaining the top K largest eigenvalues and their associated eigenvectors, the matrix is compressed, preserving critical information while drastically reducing computational resource consumption. Thus, after SOCS simplification, the TCC matrix remains vectorial and decomposed into a series of low-rank kernels $\varphi_{k}$, expressed as:

(3) $$T\mathrm{CC}\left(f^{\prime },g^{\prime };f^{\prime \prime },g^{\prime \prime }\right)=\sum_{k=1}^{K}\omega_{k}\varphi_{k}\left(f^{\prime },{\;g}^{\prime }\right)h_{k}^{*}\left(f^{\prime },{\;g}^{\prime }\right)$$

The aerial image calculation can then be expressed as:

(4) $$I\left(x,\;y\right)=\sum_{k=1}^{K}\omega_{k}|h_{k}\left(x,\;y\right)\text{ ⮾ }M\left(x,\;y\right){|}^{2}\;$$

where $h_{k}$ represents the inverse Fourier transform of $\varphi_{k}$ (the convolution kernel), $\omega_{k}$ denotes the corresponding coefficient, and ⮾ indicates the convolution operation. In our implementation, we use K = 24 for the SOCSs approximation. Finally, the aerial image *I* is converted to a binary wafer image *Z*:

(5) $$Z\left(x,\;y\right)=\left\{\begin{array}{c} 1,\;\mathrm{if}\;I\left(x,y\right)\geq I_{th}\\ 0,\;\mathrm{if}\;I\left(x,\;y\right)<I_{th} \end{array}\right.$$

where the threshold *I_th_* is set to 0.225 in our implementation. This binary process resists the constant threshold model (CTR). Beyond the constant threshold model (CTM), several other key metrics and models are essential in lithography:

NILS (Normalized Image Log-Slope): Measures the steepness of the aerial image’s intensity gradient. A higher NILS indicates a sharper light-to-dark transition, implying better potential resolution and contrast. It allows early assessment of optical image quality before exposure.

MEEF (Mask Error Enhancement Factor): Quantifies how mask-level dimensional errors are amplified onto the wafer, reflecting the process sensitivity to mask defects and manufacturing constraints.

LER (Line Edge Roughness): Evaluates the nanoscale roughness or irregularity along the edges of printed features on the wafer.

The CTM simplifies the complex photoresist chemistry into a binary physical threshold: resist dissolves where intensity exceeds the threshold (*I* > *I_th_*) and remains otherwise. Compared to the above metrics/models, the CTM is highly simplified—it ignores photoresist effects such as chemical kinetics and acid diffusion, resulting in poor prediction accuracy and making it unsuitable for advanced nodes. However, its computational efficiency and ease of implementation make it widely adopted in inverse lithography technology (ILT) research, as in this work.

### 2.2. ILT Evaluation Metrics

In ILT mask optimization, several key metrics are employed to evaluate mask quality and optimization performance. Beyond computational runtime (turnaround time), these metrics primarily assess two critical aspects: printability and manufacturability. Printability metrics include L2-norm error, process variation band (PVB), and edge placement error (EPE), while manufacturability is quantified via shot count (#Shot), representing the number of rectangular exposures required for mask fabrication.

**Definition 1**  (L2-Norm error)**.** *For a target layout Z_t_, this metric evaluates the squared Euclidean distance between the nominal wafer image Z_nom_ (generated under standard dose and focus conditions P_nom_) and Z_t_:*

(6) $$L_{L2}={\left||\;Z_{nom}-Z_{t}|\right|}_{2}^{2}$$

This quantifies global pattern fidelity, with lower values indicating better mask accuracy.

**Figure 2:**
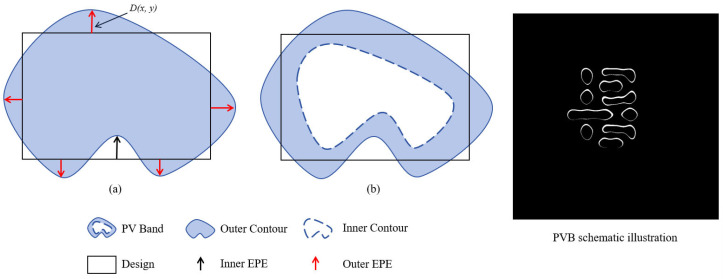
Edge placement error (EPE) and process variation band (PVB) schematic representations: (**a**) EPE; (**b**) PVB.

**Definition 2**  (Process Variation Band, PVB)**.** *As illustrated in Figure 2b, process variations (e.g., defocus and dose fluctuations) induce morphological deviations in resist patterns. PVB measures the robustness of lithographic performance by calculating the XOR area between the maximum (Z_max_) and minimum (Z_min_) wafer image contours across process conditions:*

(7) $$L_{PVB}={\left||\;Z_{max}-Z_{min}|\right|}_{2}^{2}$$

**Definition 3**  (Edge Placement Error, EPE)**.** *EPE evaluates local edge fidelity (Figure 2a). Detection points are uniformly sampled along vertical/horizontal edges, and violations are flagged when the perpendicular distance D(x,y) between target and printed edges exceeds a threshold th_EPE_. This method avoids pixel-wise computation, instead using edge-segment sampling for efficiency.*

**Definition 4**  (Mask Fracturing Shot Count, #Shot)**.** *For a given mask M, #Shot counts the minimum number of rectangular VSB (Variable Shaped Beam) exposures needed to reproduce the mask geometry with high fidelity. Fewer shots imply lower mask-writing complexity and cost.*

## 3. Methodology

This section first provides a detailed introduction to the EAAUnet network model adopted in the proposed EAAUnet-ILT scheme, including the specific functions and mechanisms of the modules contained in the model. Then, it explains the selection of optimization objectives and how to design the loss function based on these objectives, covering both the training scheme and the customized layer iteration approach. Subsequently, the paper introduces the specific details of the constraint algorithm applied to the masks generated by the EAAUnet-ILT.

**Figure 3:**
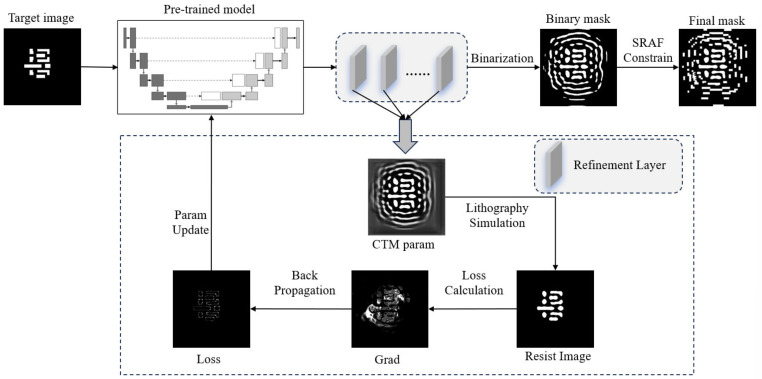
End-to-end ILT workflow of integrating a pre-trained model and customized refinement iteration.

In the complete EAAUnet-ILT workflow, we first train the model and then place the pre-trained model into customized refinement layers for iteration. These refinement layers also consist of trained EAAUnet models but differ from the pre-training phase in their loss function construction. EAAUnet-ILT represents an end-to-end ILT solution, with its workflow illustrated in Figure 3. The iteratively generated masks are Continuous Transmission Masks (CTMs), which are obtained by applying the sigmoid transformation to the intermediate parameter feature maps output by the model. The transformation formula is as follows:

(8) $$M=\frac{1}{1+e^{-\theta \times P}}$$

In the equation, *θ* represents the steepness parameter of the sigmoid function transformation, which is a hyperparameter set to 5 in this study. The iteratively generated CTM undergoes binarization processing followed by SRAF (Sub-Resolution Assist Feature) constraints to form the final mask.

### 3.1. EAAUnet Structure

**Figure 4:**
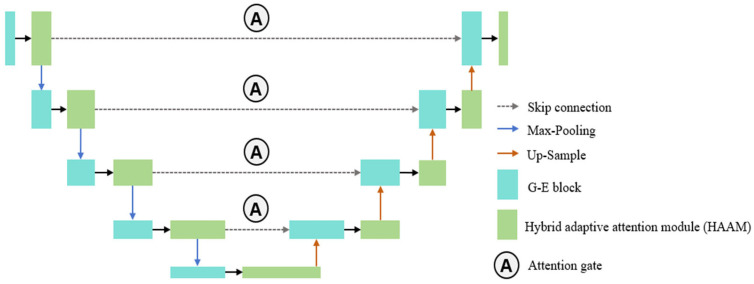
Schematic of the EAAU-net architecture. The network retains a U-shaped structure, consisting of four downsampling and four upsampling stages (five levels in total). Each level contains a Hybrid Adaptive Attention Module (HAAM) and a G-E optimized convolution module.

The EAAUnet is based on the U-Net architecture, with its overall structure illustrated in Figure 4. The number of feature map channels at each resolution level (from high to low) is set to 16, 32, 64, 128, and 256, respectively. Compared to the traditional U-Net, this adjustment reduces computational complexity.

Instead of conventional convolutional layers, EAAUnet employs Hybrid Adaptive Attention Modules (HAAMs). A key improvement lies in replacing some HAAMs with G-E modules, which maintain feature extraction capability while reducing model complexity. Additionally, attention gates [20] are incorporated into the traditional U-Net skip connections. While standard skip connections preserve low-level spatial details to compensate for spatial information loss caused by deep downsampling, the introduction of attention gates dynamically computes importance weights for encoder features, optimizing feature selection. This mechanism selectively emphasizes relevant regions while suppressing irrelevant background, avoiding redundancy from simple concatenation.

The attention gate (AG) functions as follows:

Intelligently fusing encoder features provided by HAAMs: Serving as a bridge between the encoder and decoder, the AG processes features generated jointly by the HAAMs and G-E modules in the encoder. Since these features have already undergone multi-scale extraction and attention-based filtering by HAAMs, the AG receives higher-quality encoder features, enabling more accurate fusion decisions.

Enhancing decoding precision: Through precise feature fusion, the decoder can more effectively integrate high-level semantic context with low-level detailed features during mask image reconstruction. This capability is particularly beneficial for generating accurate sub-resolution assist features (SRAFs) in critical regions such as edges and corners, directly contributing to improved final mask quality.

The collaboration within EAAUnet can be summarized as a well-defined and interlocking workflow with a clear division of labor:

HAAMs—feature mining, it is deployed at the beginning of each encoder and decoder layer. It extracts rich, multi-scale features from inputs, providing a high-quality data foundation for subsequent processing.

G-E Module—lightweight and feature enhancement, it is placed at the later stages of the encoder and decoder. It refines and enhances features extracted by HAAMs while significantly reducing parameters and computational cost, ensuring network efficiency.

Attention Gate (AG)—information fusion coordinator, it operates on skip connections to intelligently filter and fuse detailed features from the encoder with contextual features from the decoder. This ensures that the integrated information is the most relevant and effective, avoiding redundancy.

Ultimately, under the overarching symmetric architecture of U-Net, all these modules work together to achieve accurate and efficient mapping from target regions to optimized masks.

### 3.2. Hybrid Adaptive Attention Module (HAAM)

**Figure 5:**
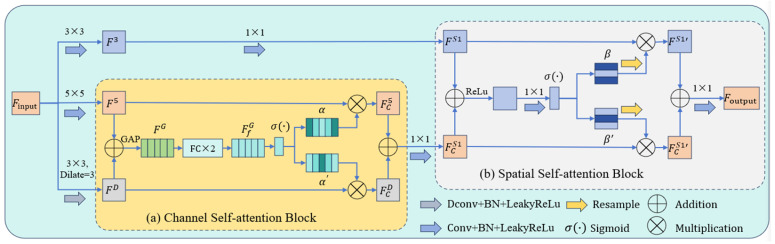
Structure of the Hybrid Adaptive Attention Module (HAAM): (**a**) channel self-attention block; (**b**) spatial self-attention block.

The HAAM enhances feature extraction by adaptively selecting features through multi-scale convolution and a dual-attention mechanism (channel attention and spatial attention). Its detailed structure is shown in Figure 5. The HAAM consists of three components: multi-scale convolutional layers with different kernel sizes, a channel self-attention block, and a spatial self-attention block. The input feature $F_{input}$ is first processed by three parallel convolutional layers, generating feature maps with different receptive fields: $F^{3}$: Obtained via a 3 × 3 convolution, $F^{5}$: Obtained via a 5 × 5 convolution, and $F^{D}$: Obtained via a 3 × 3 dilated convolution (dilation rate = 3). These three convolutions capture features at different scales: The 5 × 5 convolution provides a receptive field equivalent to two stacked 3 × 3 convolutions, The dilated convolution expands the receptive field to that of five 3 × 3 convolutions, enhancing multi-scale feature extraction.

#### 3.2.1. Channel Self-Attention Block

The channel self-attention block is designed to extract the most effective features from feature maps with receptive fields of varying sizes. As illustrated in Figure 5a, this block recalibrates channel feature responses by modeling interdependencies among channels, thereby screening out the most representative features. First, global average pooling (GAP) compresses $F^{5}\in R^{C\times H\times W}$ and $F^{D}\in R^{C\times H\times W}$ into a new feature $R^{2C\times 1\times 1}$ with dimensions 1 × 1:

(9) $$F^{G}=GAP\left(F^{5}+F^{D}\right)$$

The feature map $F^{G}$ is then fed into two cascaded fully connected layers, followed by a batch normalization layer and a ReLU activation layer, to generate a new feature map:

(10) $$F_{f}^{G}=\sigma_{ReLu}\left(BN\left(\omega_{fc1}\omega_{fc2}{\;\cdot \;F}^{G}\right)\right)$$

Subsequently, the sigmoid activation is applied to $F_{f}^{G}$ to normalize the weights of each channel into probabilistic form, yielding the channel attention map:

(11) $$\alpha =\sigma \left(F_{f}^{G}\right)$$

Here, $\alpha \in {\left[0,\;1\right]}^{C\times 1\times 1}$ and $\alpha^{\prime }\in {\left[0,\;1\right]}^{C\times 1\times 1}$ represent the channel attention maps for $F^{D}$ and $F^{5}$, respectively, with each value indicating the importance of the corresponding channel in the feature map. $\alpha^{\prime }$ is derived from α as 1−α. The feature maps are then calibrated using the channel attention maps:

(12) $$F_{C}^{D}=\alpha \text{⮾}F^{D}$$

(13) $$F_{C}^{5}=\alpha^{\prime }\text{⮾}F^{5}$$

#### 3.2.2. Spatial Self-Attention Block

The spatial self-attention block focuses on the spatial locations of features, taking as input the features from 3 × 3 convolutions and the output feature maps from the channel self-attention block, as shown in Figure 5b. First, a 1 × 1 convolution is applied to the input feature maps to facilitate the subsequent processing of spatial information:

(14) $$F^{S1}={Conv}_{1\times 1}\left(F^{3}\right)$$

(15) $$F_{C}^{S1}={Conv}_{1\times 1}\left(F_{C}^{D}+F_{C}^{5}\right)$$

The convolved features $F^{S1}$ and $F_{C}^{S1}$ are summed, then processed through ReLU activation $\sigma_{ReLu}(\cdot )$, 1 × 1 convolution, and sigmoid activation σ(·) to obtain the spatial attention map *β*:

(16) $$\beta =\sigma ({Conv}_{1\times 1}\left({\sigma_{ReLu}(F}^{S1}+F_{C}^{S1}\right)))$$

Here, β∈[0, 1] denotes the spatial attention map for $F_{C}^{S1}$, while $\beta^{\prime }\in [0,\;1]$ represents the spatial attention map for $F^{S1}$, with $\beta^{\prime }=1-\beta$. β and $\beta^{\prime }$ resampled, and the resampled versions are used to calibrate the feature maps, yielding $F_{C}^{S1\prime }$ and $F^{S1\prime }$, respectively. Finally, the two are summed up and convolved to produce the output $F_{output}$:

(17) $$F_{output}={Conv}_{1\times 1}\left(F^{S1\prime }+F_{C}^{S1\prime }\right)$$

In summary, the HAAM performs as follows:

Providing preprocessed rich features for the G-E module: Positioned at the front end of each level, the HAAM performs preliminary yet powerful feature extraction and filtering. It delivers the processed feature maps ($F_{output}$), rich in multi-scale information, to the G-E module at the same level. This allows the G-E module to perform subsequent lightweight operations based on higher-quality features, achieving better results with greater efficiency.

Supplying high-quality encoder features for the attention gate: The features extracted by HAAM in the encoder path are transmitted to the decoder via skip connections. These features, refined by the attention mechanism, contain more effective information, laying a solid foundation for the precise decision-making of the attention gate.

### 3.3. G-E Module

**Figure 6:**
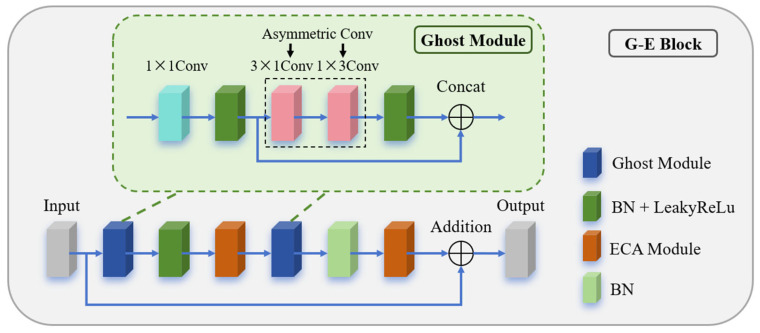
Structure of the G-E module.

The G-E module is a lightweight block designed to enhance feature extraction capabilities while minimizing parameter count and computational resource consumption. As illustrated in Figure 6, the G-E block consists of a main path and a shortcut path. The main path incorporates a Ghost module [21] and an ECA module [22]. Compared to traditional convolution, the Ghost module significantly reduces computational costs by leveraging feature redundancy and channel correlations to generate effective features. The ECA module further enhances the feature maps from the Ghost module through adaptive channel calibration, enabling the module to focus more on critical information while maintaining low computational overhead and improving ILT performance.

**Figure 7:**
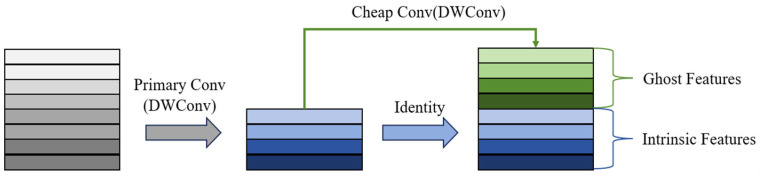
Mechanism of the Ghost module.

Figure 7 illustrates the mechanism of the Ghost module, which simplifies traditional convolution via a three-stage hierarchical decomposition. First, pointwise convolution compresses the input Finput along channels to extract intrinsic feature representations. Next, two consecutive asymmetric convolutions (a combination of 3 × 1 and 1 × 3 convolutions, denoted as AsymConv) replace depthwise convolution to generate additional features, termed “ghost features,” based on these intrinsic representations. Finally, the ghost features are concatenated with the intrinsic features to form a composite tensor FGhost1, produced by the first Ghost module. The computation process is described by Equations (18)–(20):

(18) $$F_{prim\;conv}=BN(\mathrm{LReLU}\left(\mathrm{PWConv}(F_{input}\right)))$$

(19) $$F_{cheap\;op}=BN(\mathrm{LReLU}\left(\mathrm{DWConv}(F_{prim\;conv}\right)))$$

(20) $$F_{Ghost1}=\mathrm{Concat}(F_{prim\;conv},\;{\;F}_{cheap\;op})$$

After batch normalization (BN) and LeakyReLU (LReLU) activation, $F_{Ghost1}$ is mapped to a dimension-preserved tensor *P*, which is then processed by the ECA module for global text-aware channel adaptation. The equation is as follows:

(21) $$P=\mathrm{BN}\left(\mathrm{LReLU}(F_{Ghost1})\right)$$

The tensor *P* undergoes squeeze-and-excitation operations in the ECA module, eliminating the side effects of dimensionality reduction in SE blocks. A 1D convolution captures inter-channel relationships, with the kernel size k automatically determined based on the number of channels. The output is a channel-attention-enhanced feature map $F_{ECA1}$. An additional Ghost module is appended after the ECA module to compress the channel dimensions of $F_{ECA1}$, further reducing computational costs. Notably, only batch normalization (BN) is applied after the second Ghost module, omitting LReLU activation to prevent excessive information loss due to reduced channel dimensions. The second ECA module yields another channel-attention-enhanced feature map $F_{ECA2}$.

Meanwhile, the initial feature map on the shortcut path undergoes depthwise separable convolution to produce $F_{initial}$, aligning its channel count with the output of the main path. The final output of the G-E module is obtained by element-wise addition of $F_{ECA2}$ and $F_{initial}$. This approach preserves original information without altering feature dimensions or introducing extra parameters, ensuring low computational complexity.

In summary, The G-E block functions as follows:

It receives and refines the output from the HAAM: Positioned right after HAAM, the G-E module takes in the multi-scale features that have been preliminarily filtered by the HAAM’s attention mechanism. It then performs two key operations:

Ghost operation: Significantly reduces the channel dimensionality and computational cost of the feature maps, achieving model lightweighting.

ECA attention: Further “purifies” the compressed features at the channel level, ensuring that critical information is preserved and even enhanced.

It supplies efficient features to the next layer: The processed features retain high representational capacity while being highly lightweight. These features are passed to the next downsampling or upsampling layer, enabling efficient information propagation throughout the deep network.

### 3.4. Model Pretrain and Iteration

The proposed EAAUnet-ILT framework employs a two-stage process: (1) pretraining via supervised learning to improve convergence efficiency, and (2) refinement training for precise pattern optimization. The pretraining phase uses a composite loss function derived from lithography performance metrics:

(22) $$L_{pre}=L_{L2}+L_{PVB}+L_{label}$$

(23) $$L_{label}={\left||M-M_{label}^{*}|\right|}_{2}^{2}$$

Here, $L_{label}$ is a constraint term ensuring rapid convergence by steering the model’s output mask M toward the target mask $M_{label}^{*}$. After pretraining, the EAAUnet model undergoes refinement training with customized layers to further enhance ILT performance. The refinement loss function is defined as:

(24) $$L_{refine}=L_{L2}+L_{PVB}+{\gamma \;\cdot \;L}_{regul}$$

(25) $$L_{regul}=e^{-\frac{k\;\cdot \;n}{N}}{\left||P|\right|}_{2}^{2}$$

where $L_{regul}$ is a regularization term with exponential decay, and *k* is a decay weight, *N* is the total number of refinement layers (iterations), and n is the current refinement layer (iteration). This strategy helps avoid overfitting, escape local optima, and improve mask optimization. As refinement progresses, the regularization coefficient γ approaches zero, preventing stagnation in local optima. To achieve this effect—specifically decaying to one-thousandth of its initial value—the decay weight k is set to ln(1000)= 6.9. The complete EAAUnet-ILT framework integrates these steps, as detailed in Algorithm 1.
**Algorithm 1** EAAUnet-ILT Application Process**Require:** target layout $Z_{t}$, labeled mask $M_{label}^{*}$, kernels $h_{k}$, dose d, weight $\omega_{k}$, pretrained model EAAUnet_model**Ensure:** SRAF constrain mask $M_{con}$
1:   **function** GMUNet -ILT($Z_{t}$, $h_{k}$, d, $\mu_{k}$) 2:       load_model(EAAUnet_model)3:           **for** i = 1, …, *th*_iter_4:              M ← EAAUnet($Z_{t}$)5:              Z ← litho_sim(M)6:              $L_{refine}$ ← calculate_loss(Z, $Z_{t}\;$, $M_{label}^{*}$)7:              G ← calculate_gradient(L)8:              update parameters(EAAUnet_model)9:           **end for**10:         $M_{binary}\leftarrow \mathrm{Binary}(M)$11:         $M_{con}\leftarrow \mathrm{SRAF}\text{\_}\mathrm{constrain}(M_{binary})$12:      **return** $M_{con}$
13:  **end** function

### 3.5. SRAF Constrain Algorithm

The mask M output by EAAUnet-ILT contains irregular and complex SRAFs. Considering the manufacturability of the mask, we employ a rectangular decomposition-based constraint algorithm to regulate the SRAF patterns. Specifically, given the binary mask $M_{binary}$ and the target pattern $Z_{t}$, we first dilate the contour of $Z_{t}$ by a certain distance to create a protected region $M_{P}$. This protected region envelops the main pattern of $M_{binary}$ while preserving the original features within it. The remaining region $M_{R}$ is used to extract SRAFs for constraint operations.

First, portions of $M_{R}$ exceeding a certain threshold are extracted as the SRAF constraint region $M_{SRAF}$. $M_{SRAF}$ is then segmented into connected components, each of which undergoes grid decomposition. Rectangles are generated based on the decomposed grids by setting all pixel values within each grid to 1. If a connected component is too small, rectangles are directly generated according to the minimum size constraint. The formulation is as follows:

(26) $$W_{grid}=min(S_{max},max(S_{min},\;w))$$

(27) $$H_{grid}=min(S_{max},\;max(S_{min},\;h))$$

(28) $$W_{step}=max(1,\;W_{grid}\;\cdot \;(1-r))$$

(29) $$H_{step}=max(1,\;H_{grid}\;\cdot \;(1-r))$$

Here, $W_{grid}$ and $H_{grid}$ denote the width and height of the grid, respectively; $S_{max}$ and $S_{min}$ represent the maximum and minimum allowable side lengths of the rectangles; w and h correspond to the width and height of the extracted connected component; $W_{step}$ and $H_{step}$ are the overlap dimensions, meaning generated rectangles overlap by these parameters; and r is the lower bound for the pixel percentage of the constrained SRAF pattern relative to the originally extracted SRAF region in the CTM, ensuring the coverage of the constrained pattern exceeds this value. A higher r makes the result closer to the original SRAF pattern, but an excessively high value may compromise algorithmic efficiency—hence, we set r = 0.5 in this work.

**Figure 8:**
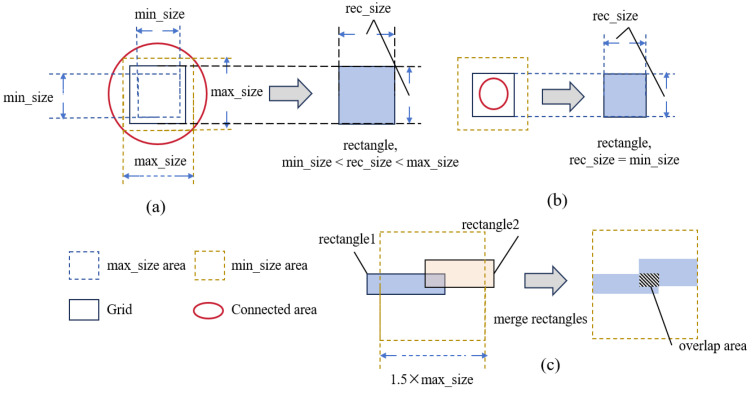
Mechanism of the SRAF constraint algorithm: (**a**) when the grid size of a connected component lies between the maximum and minimum size limits, the generated rectangle matches the grid dimensions; (**b**) if a connected component is too small to accommodate the minimum grid size, the generated rectangle is fixed as a square with the minimum allowable size; (**c**) when the maximum axial distance between two merged rectangles in any direction exceeds 1.5× the maximum size limit, the merged rectangle is constrained within the permissible range.

After generating the rectangles, they are merged to reduce the complexity of the SRAF pattern. The merged rectangles must still satisfy size constraints, with the maximum axial pixel distance difference between merged patterns in vertical or horizontal directions not exceeding 1.5 × $S_{max}$. Finally, the merged rectangles are combined with the protected region M_P to form the complete mask. Figure 8 illustrates the rules for rectangle generation and merging based on grids, and Algorithm 2 outlines the SRAF constraint process.
**Algorithm 2** Mask SRAF Constrain**Require:** Binary mask $M_{binary}$, target layout $Z_{t}$, max size $S_{max}$, min size $S_{min}$, overlap ratio ***r*****Ensure:** Constrained mask $M_{con}$
1:    **function** SRAF_constrain($M_{binary}$, $Z_{t}$, $S_{max}$, $S_{min}$, ***r***)2:       $M_{P}\leftarrow {dilate(Z}_{t})$3:       $M_{R}\leftarrow M_{binary}\;not\;M_{P}$4:       {$M_{1}$, …, $M_{n}$} ← findConnectedRegions($M_{R}$)5:           **for** i = 1, …, n6:                   $G_{i}$ ← getGrid($M_{i}$)7:                   $R_{i}$ ← generateRectangle($G_{i}$)8:           **end for**9:           $M_{SRAF}$ ← mergeRectangles($R_{1}$, …, $R_{n}$)10:         $M_{con}$ ← $M_{SRAF}$ **or** $M_{P}$
11:         **return** $M_{con}$
12:  **end** function

## 4. Experiments and Discussion

### 4.1. Experimental Materials

The proposed EAAUnet-ILT framework is implemented using PyTorch 2.4.1 with CUDA acceleration and runs on a Linux-based computing platform equipped with a 2.6 GHz Intel Xeon processor and an NVIDIA RTX 4090 GPU. Performance benchmarking was conducted using the ICCAD 2013 CAD Contest benchmark suite [23], which includes ten 2048 × 2048 industrial-grade M1 layer designs at the 32 nm technology node, along with its integrated lithography simulation engine. For the ICCAD 2013 lithography simulation engine: Wavelength: 193 nm; NA: 1.35; Partial coherence factors: the inner σ = 0.3, the outer σ = 0.9; Annular illumination (exact parameters unspecified in documentation/engine code); Pupil apodization (PSF) corresponds to optical kernel $h_{k}$ in Equation (4), with $\omega_{k}$ as the weight; Polarization: TE (electric field perpendicular to the plane of incidence) and TM (magnetic field perpendicular to the plane of incidence); Imaging grid resolution: 1 nm/pixel; Mask type: binary; Photoresist model: constant threshold resist (CTR), details provided in Section 2.1. It should be noted that mask binarization was implemented according to Equation (5), without employing any smoothing techniques prior to binarization. The binarization threshold was set to 0.5. Since the CTM is a continuous pixel matrix constrained by a steep sigmoid function (making it nearly binarized), and the lithography simulation uses the CTM for the final binarization of the aerial image results, the choice of binarization threshold has no impact on the lithography simulation or ILT outcomes.

The training dataset, provided by the authors of GAN-OPC [24], consists of 4875 M1 layer layout patterns compliant with 32 nm design rules. For PV band computation, an exposure dose range of ±2% and a defocus range of ±25 nm is considered. Actually, we only consider three scenarios for the process window grid: standard dose and focus condition, +2% dose and +25 nm defocus condition, −2% dose and −25 nm defocus condition, which is also configured according to the protocols adopted by most studies [12,24,25], aiming to simplify the experimental variables and facilitate the quantitative comparison of PVB metrics. The coefficient of regularization term is set to 0.3, and for the SRAF constrain algorithm, max_size is set to 36 nm and min_size is set to 20 nm.

**Figure 9:**
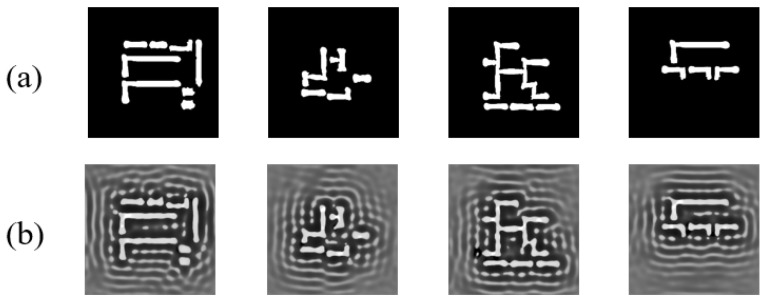
Illustration of label datasets: (**a**) original label data from GAN-OPC [24]; (**b**) CTM label data generated via our gradient descent optimization.

To enhance training effectiveness, we followed the approach in UNeXt-ILT [26] by replacing the original labels in the GAN-OPC dataset with CTM (Continuous Transmission Mask) labels generated via gradient descent, incorporating SRAF patterns. As illustrated in Figure 9, Figure 9a shows the original label mask, while Figure 9b presents our gradient-descent-generated label mask. During both CTM label generation and model inference/refinement, all test target layouts are resized to 1024 × 1024 to balance optimization effectiveness and computational efficiency. While larger lithography simulations improve accuracy, they require excessive time, whereas smaller layouts significantly reduce precision. For CTM label generation via gradient descent: 10 iterations with step size 0.5; input training images are smoothed using Equation (8) (steepness = 4.0) to facilitate differentiation; the loss function matches Equation (24) with identical regularization; SGD optimizer with γ = 0.3. For EAAUnet refinement: Adam optimizer (initial learning rate = 2 × 10^−3^), step decay scheduler (step size = 7 epochs, decay factor = 0.1), batch size = 2. The iteration count was experimentally determined, as discussed later.

**Figure 10:**
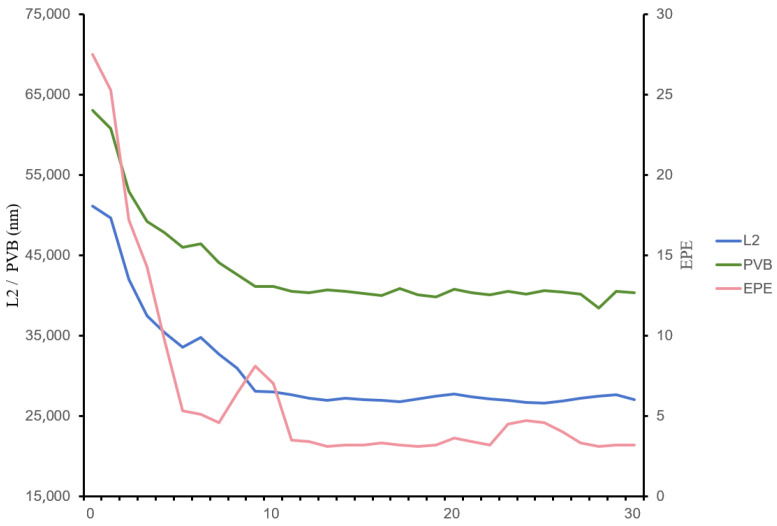
Schematic diagram of the variation trends of average key ILT metrics with model iteration.

To determine the appropriate number of iterations, we initially set the regularization coefficient in the loss function (Equation (24)) for model refinement to a relatively small yet moderate value of 0.2 to observe the convergence trend. Since the number of iterations and the strength of regularization are highly coupled, strong regularization tends to force the loss function to reach a plateau more quickly. However, this may result from excessive suppression by regularization rather than indicating a truly optimal solution. Therefore, we first employe weak regularization to explore and understand the inherent optimization difficulty of the primary loss function itself. We first investigated the relationship between the number of iterations and average ILT metrics (L2, PVB, EPE) of the ten benchmarks. As shown in Figure 10, after the number of iterations reaches 10, the metrics generally stabilize—with the exception of minor fluctuations in EPE—indicating that the model parameters have also reached a convergent state. Based on the observed trend, we set the model refinement iteration count to 15. After establishing the suitable iteration count, we proceeded to conduct ablation studies on the regularization coefficient in the work presented in Section 4.3.

**Figure 11:**
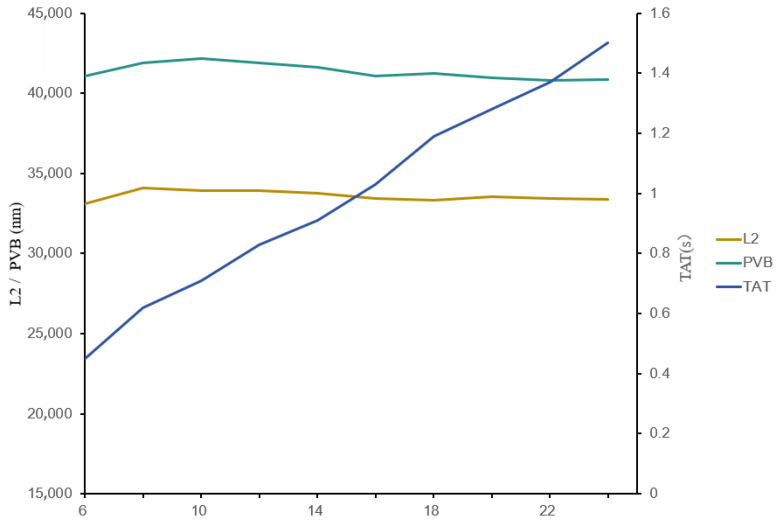
Schematic diagram of the impact of SOCS order K on lithography performance metrics.

We also analyzed the SOCS approximation order in a Hopkins equation-based lithography system, testing orders from K = 6 to K = 24 in steps of two. The results (Figure 11) show that the order has no significant impact on lithographic metrics, confirming the effectiveness and rationality of SOCSs—even a low order (K = 6) sufficiently approximates the complex TCC-based Hopkins model. Moreover, as the approximation order decreases, the model runtime is nearly reduced linearly. However, to align with the widely prevalent use of K = 24 in current research, we retain this setting for fair comparison.

**Figure 12:**
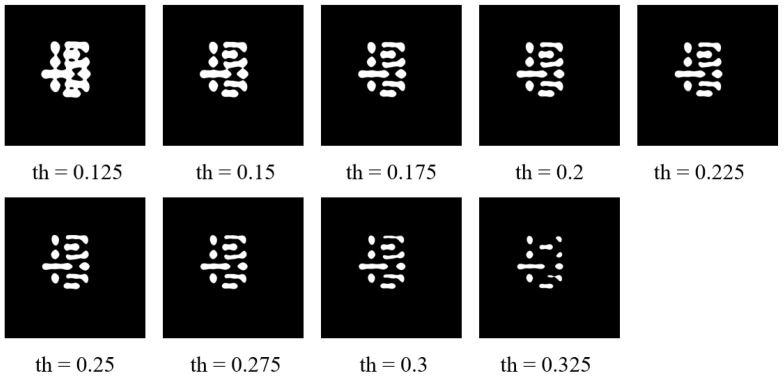
Impact of different photoresist imaging thresholds on simulated wafer patterns in lithography.

Finally, we experimentally investigated the imaging threshold of the photoresist CTM. The threshold was adjusted from 0.125 to 0.325, and the results are summarized in Table 1 and Figure 12. From Figure 12, as the threshold increases, the lithographic pattern develops more extensively, resulting in larger and wider features (i.e., larger CD); conversely, lower thresholds lead to less development and narrower lines (i.e., smaller CD). Correspondingly, Table 1 shows that when the threshold is either too high or too low, the L2 error and EPE increase significantly due to substantial deviation in the contour. In contrast, the PVB decreases under these conditions, since over- or under-development limits the sensitivity of the contour to process variations. Furthermore, it can be observed that a threshold within the range of 0.21–0.24 ensures relatively stable lithography quality, and 0.225 is identified as the optimal value within this range, yielding the best overall performance metrics.

### 4.2. Results and Analysis

First, to validate the effectiveness of the improvements in the adopted EAAUnet compared to the original AAUnet architecture, we designed controlled experiments with the following variants:

1. AAUnet enhanced with an attention gate (AG) (i.e., EAAUnet without the G-E block improvement);
2. AAUnet enhanced with a G-E block (i.e., EAAUnet without the attention gate mechanism);
3. AAUnet with a modified G-E block where the ghost module was replaced with a standard convolution and the ECA module was removed (denoted as variant0 in Table 1).

Additionally, to demonstrate the effectiveness of the HAAM in the original model, we conducted comparative experiments between a pure standard convolutional structure (conv stage, equivalent to U-Net), and AAUnet (where the HAAM replaces part of the standard convolutions). The results are summarized in Table 1, in which TAT represents the average inference time of the ten benchmark single tiles.

The data in Table 2 demonstrate that our enhanced EAAUnet achieves comprehensive optimization in ILT performance, runtime, and model parameter efficiency, resulting in a more lightweight yet feature-rich model.

Next, we compared EAAUnet-ILT with state-of-the-art (SOTA) ILT methods, including Neural-ILT [25], A2-ILT [27], and Multi-ILT [12]. Table 3 and Table 4 present comparisons in terms of L2 squared error, PV band (Process Variation Band), and turnaround time, while Table 5 compares EPE (Edge Placement Error) and Mask Fracturing Shot Count (#Shot).

**Figure 13:**
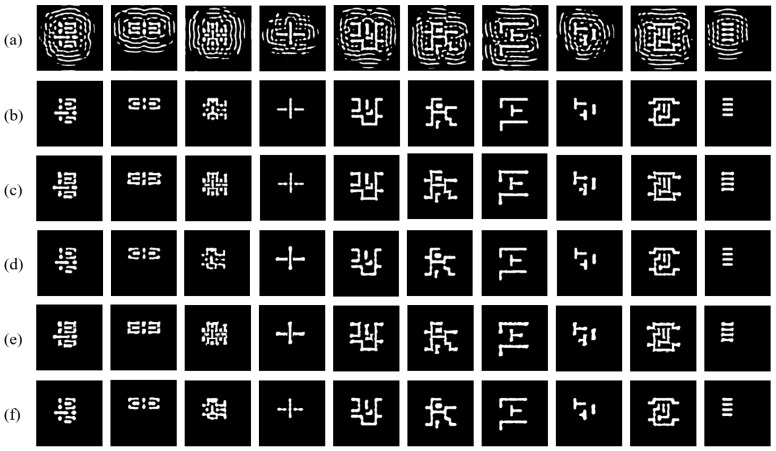
Visual comparison of the mask patterns and simulated wafer prints across different ILT methods: (**a**) masks of ours; (**b**) wafer images of (**a**); (**c**) masks of Neural-ILT [25]; (**d**) wafer images of (**c**); (**e**) masks of A2-ILT [27]; (**f**) wafer images of (**e**).

Compared to these SOTA ILT methods, our EAAUnet-ILT achieves superior performance across all metrics—L2 error, PV band, EPE, and runtime—while our proposed SRAF constraint scheme significantly improves mask manufacturability. Specifically, against Neural-ILT, EAAUnet-ILT reduces L2 error by 39%, PV band by 27%, EPE by 36%, and 6.13× speedup; compared with A2-ILT, it reduces L2 error by 36%, PV band by 26%, and 3.02× speedup; compared with Multi-ILT, it reduces L2 error by 6%, PV band by 5%, and 1.77× speedup, while all referenced comparative schemes have open-source implementations, which we executed on our hardware and platform with their configurations preserved intact. Furthermore, we provide a visual comparison between EAAUnet-ILT and several state-of-the-art (SOTA) ILT methods on benchmark cases, as shown in Figure 13. Figure 13a displays the mask generated by EAAUnet-ILT, while Figure 13b presents the lithography simulation result of this mask. Figure 13c shows the mask generated by Neural-ILT, with its corresponding lithography simulation result in Figure 13d. Similarly, Figure 13e illustrates the mask produced by A2-ILT, and Figure 13f demonstrates the lithography simulation outcome of this mask. As evident from Figure 13, our EAAUnet-ILT method generates masks with stable and high-quality multi-loop Sub-Resolution Assist Features (SRAF), and the resulting wafer image from lithography simulation also achieves the highest quality.

Furthermore, with the SRAF constraint scheme applied, EAAUnet-ILT achieves an average 76% reduction in #Shot while incurring only a 12% degradation in lithographic L2 performance and a 9% loss in PV band performance. The runtime remains competitive, with the SRAF constraint algorithm accounting for 21% of the total computation time, demonstrating its feasibility and superiority. Table 6 presents the runtime breakdown by stage for EAAUnet-ILT.

### 4.3. Ablation Study

**Figure 14:**
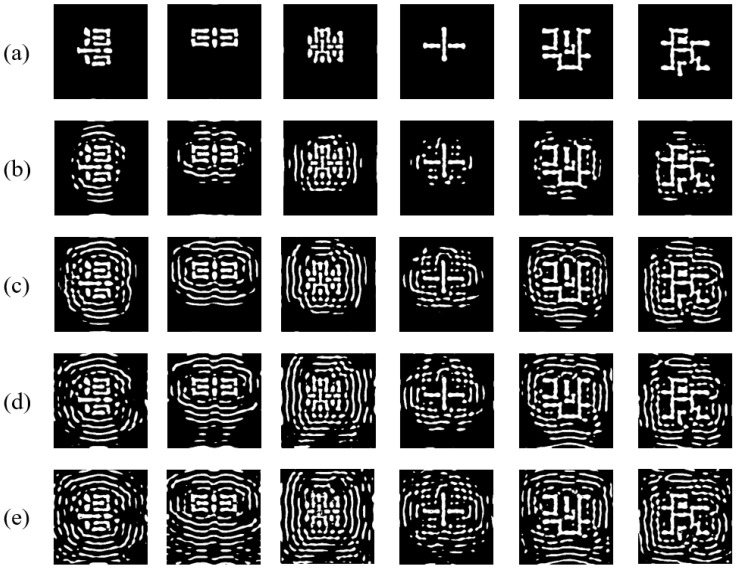
Comparison of masks generated by EAAUnet-ILT under different regularization coefficients: (**a**) masks of γ = 0; (**b**) masks of γ = 0.1; (**c**) masks of γ = 0.2; (**d**) masks of γ = 0.3; (**e**) masks of γ = 0.4.

To further validate the effectiveness of the regularization term and the SRAF constraint algorithm, we conducted ablation experiments on EAAUnet-ILT, examining the impact of adjusting the regularization coefficient and SRAF constraint parameters. Table 7 quantifies the influence of different regularization coefficients on ILT performance when the SRAF constraint is disabled. Figure 14 illustrates the mask patterns generated under varying regularization strengths, showing that higher γ values lead to more SRAFs and more complex mask geometries. While increasing γ within a certain range reduces L2 error and PV band, excessive values hinder optimization. Thus, we set γ = 0.3 in this work, same as the CTM label gradient descent generation.

**Figure 15:**
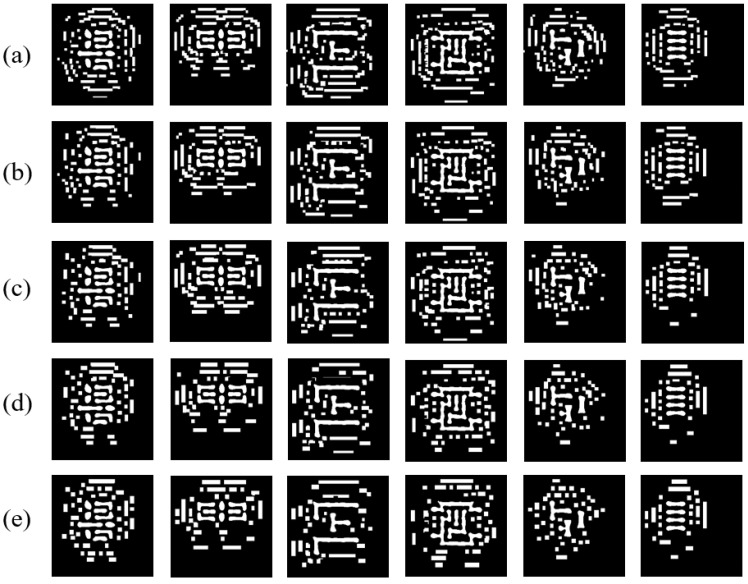
Comparison of constrained SRAF patterns under different input parameters: (**a**) masks of min_size = 12 nm, max_size = 28 nm; (**b**) masks of min_size = 16 nm, max_size = 32 nm; (**c**) masks of min_size = 20 nm, max_size = 36 nm; (**d**) masks of min_size = 24 nm, max_size = 40 nm; (**e**) masks of min_size = 28 nm, max_size = 44 nm.

Table 8 and Table 9 jointly analyze the effect of SRAF constraint parameters on mask quality. Results indicate that smaller constraint sizes yield masks closer to the original unconstrained version, improving ILT metrics at the cost of higher #Shot (reduced manufacturability) and longer runtime. Figure 15 visually demonstrates how constraint size affects SRAF patterns—smaller constraints produce finer, more densely packed rectangles that better approximate the original SRAFs.

## 5. Conclusions

We proposed EAAUnet-ILT, a deep learning-based ILT framework built on an improved lightweight model. By leveraging spatial and channel self-attention mechanisms and optimized convolutional modules, the model enhances feature extraction while reducing computational overhead and accelerating inference. Pretrained model fine-tuning enables efficient mask optimization for target layouts. Additionally, regularization techniques dynamically adjust SRAF generation, further improving optimization. Our SRAF constraint algorithm significantly enhances mask manufacturability without compromising lithographic performance. Experimental results confirm that this ILT flow, combined with the SRAF constraint scheme, generates high-quality masks efficiently while ensuring manufacturability.

## Figures and Tables

**Table 1:** Performance comparison between different resist threshold in ILT metrics.

Resist Threshold	L2 Error (nm^2^)	PV Band (nm^2^)	EPE
0.125	97,263	30,366	107.9
0.15	53,813	32,728	31.2
0.175	42,968	35,720	23.1
0.2	31,071	38,553	8.2
0.21	28,479	39,628	8.0
0.22	27,392	40,407	7.6
0.225	27,033	40,265	7.5
0.23	27,429	41,267	7.5
0.24	28,232	42,255	7.6
0.25	29,211	43,138	8.3
0.275	43,641	48,312	10.8
0.3	62,445	45,396	22.6
0.325	113,263	39,498	54.4

**Table 2:** Performance comparison between the improved EAAUnet, the original AAUnet, and other AAUnet variants in ILT metrics.

ILT Model	L2 Error (nm^2^)	PV Band (nm^2^)	TAT (s)	GFLOPs	Model Params (K)
U-Net (Conv stage)	42,186	51,453	3.89	770.49	31,042
AAUnet (HAAM)	37,013	45,964	2.21	282.75	10,493
AAUnet + AG	36,147	45,374	2.22	284.42	10,611
Variant0	33,911	44,276	1.73	272.89	8497
AAUnet + G-E block w/o AG	27,569	40,837	1.51	231.37	7629
EAAUnet	**27,033**	**40,265**	**1.50**	224.16	**7520**

**Table 3:** Performance comparison of EAAUnet-ILT (with SRAF constraint) vs. SOTA ILT methods in L2, PVB, and runtime.

Bench-Marks	Neutal-ILT [25]			A2-ILT [27]			Multi-ILT [12]		
ID	L2	PVB	TAT	L2	PVB	TAT	L2	PVB	TAT
Case 1	49,817	55,975	10.67	45,287	59,940	4.53	40,779	50,661	2.61
Case 2	38,174	37,160	12.03	34,044	51,988	4.52	34,201	44,322	2.71
Case 3	89,411	74,387	9.76	92,505	91,261	4.56	66,486	71,527	2.68
Case 4	16,744	23,357	9.33	21,644	29,017	4.48	10,942	21,500	2.59
Case 5	45,598	48,686	6.37	38,082	61,601	4.61	30,231	51,277	2.68
Case 6	43,836	42,673	6.44	42,068	53,620	4.50	30,741	44,982	2.72
Case 7	20,324	35,862	8.77	21,947	49,053	4.56	17,101	40,294	2.63
Case 8	13,337	18,001	8.87	15,668	23,853	4.48	11,935	20,357	2.61
Case 9	49,401	56,867	10.32	46,973	68,442	4.55	35,805	57,930	2.62
Case 10	8511	15,305	9.40	10,450	19,950	4.50	8825	18,470	2.64
Average	37,515	50,964	9.20	36,867	50,873	4.53	28,705	42,132	2.65
Ratio	1.39	1.27	6.13	1.36	1.26	3.02	1.06	1.05	1.77

**Table 4:** Performance comparison of EAAUnet-ILT (with SRAF constraint) vs. SOTA ILT methods in L2, PVB, and runtime.

Bench-Marks	EAAUnet-ILT			EAAUnet-ILT+ SRAF Constrain		
ID	L2	PVB	TAT	L2	PVB	TAT
Case 1	37,872	45,110	1.50	40,996	47,131	1.81
Case 2	28,850	38,490	1.48	31,725	41,477	1.79
Case 3	65,601	72,100	1.50	67,616	77,111	1.82
Case 4	12,542	22,319	1.51	17,525	29,442	1.85
Case 5	27,668	49,799	1.49	31,230	52,366	1.82
Case 6	28,918	44,730	1.52	33,038	48,606	1.81
Case 7	10,646	37,494	1.54	12,991	39,839	1.78
Case 8	13,697	20,310	1.46	17,573	24,872	1.84
Case 9	35,237	55,033	1.56	38,236	57,908	1.83
Case 10	9295	17,266	1.44	11,745	21,390	1.85
Average	**27,033**	**40,265**	**1.50**	30,268	44,014	1.82
Ratio	**1**	**1**	**1**	1.12	1.09	1.21

**Table 5:** Performance comparison of EAAUnet-ILT (with SRAF constraint) vs. SOTA ILT methods in EPE and #Shot.

Bench-Marks	Neutal-ILT [25]		A2-ILT [27]		Multi-ILT [12]		EAAUnet-ILT		EAAUnet-ILT+ SRAF Constrain	
ID	EPE	#Shots	EPE	#Shots	EPE	#Shots	EPE	#Shots	EPE	#Shots
Case 1	8	428	— *	304	3	385	8	428	4	304
Case 2	3	256	—	258	2	284	3	256	1	258
Case 3	52	557	—	493	22	316	52	557	41	493
Case 4	2	136	—	218	0	241	2	136	2	218
Case 5	3	380	—	351	0	411	3	380	0	351
Case 6	5	383	—	301	0	415	5	383	0	301
Case 7	0	244	—	245	0	382	0	244	0	245
Case 8	0	285	—	177	0	271	0	285	0	177
Case 9	2	444	—	382	0	490	2	444	0	382
Case 10	0	208	—	152	0	164	0	208	0	152
Average	7.5	332	—	288	2.7	336	7.5	332	4.8	288
Ratio	2.34	0.20	—	0.17	0.84	0.20	2.34	0.20	1.5	0.17

* Metric data are not reported in original paper.

**Table 6:** Schematic illustration of the runtime breakdown by stage in EAAUnet-ILT.

Stage	Time Cost(s)
Model inference	0.102
Lithography simulation	0.00176
SRAF constraint	0.32

**Table 7:** Impact of different regularization coefficients on EAAUnet-ILT’s L2 and PVB metrics.

Regularization Adjust	L2 Error (nm^2^)	PV Band (nm^2^)
γ = 0	29,564	45,244
γ = 0.1	28,317	41,593
γ = 0.2	27,431	39,315
γ = 0.3	27,033	38,965
γ = 0.4	27,632	39,158

**Table 8:** Impact of SRAF constraint parameters on ILT performance.

Benchmarks	Min_Size = 12, Max_Size = 28				Min_Size = 16, Max_Size = 32				Min_Size = 20, Max_Size = 36			
ID	L2	PVB	#Shots	TAT	L2	PVB	#Shots	TAT	L2	PVB	#Shots	TAT
Case 1	37,863	45,343	544	1.85	39,005	46,950	513	1.83	40,996	47,131	450	1.79
Case 2	28,557	37,517	488	1.85	29,450	38,120	443	1.85	31,725	41,477	425	1.80
Case 3	75,759	68,001	658	1.91	76,659	73,686	627	1.87	67,616	77,111	561	1.80
Case 4	12,495	25,452	334	1.89	13,329	26,323	318	1.87	17,525	29,442	308	1.79
Case 5	29,034	50,212	596	1.90	30,889	50,574	574	1.86	31,230	52,366	537	1.86
Case 6	29,167	44,460	578	1.87	30,940	45,678	553	1.90	33,038	48,606	489	1.88
Case 7	13,933	39,973	583	1.89	14,493	38,376	569	1.81	15,991	39,839	453	1.83
Case 8	10,867	21,274	477	1.89	12,083	22,192	456	1.75	14,573	24,872	402	1.82
Case 9	36,807	57,131	809	1.86	38,084	57,725	796	1.77	38,236	57,908	651	1.87
Case 10	10,013	18,566	434	1.84	10,039	20,087	411	1.83	11,745	21,390	317	1.85
Average	27,853	40,502	550	1.87	29,497	41,971	526	1.83	30,268	44,014	459	1.82
Ratio	1.03	1.04	0.33	1.25	1.09	1.08	0.32	1.22	1.12	1.13	0.28	1.21

**Table 9:** Impact of SRAF constraint parameters on ILT performance.

Benchmarks	Min_Size = 24, Max_Size = 40				Min_Size = 28, Max_Size = 44				NO SRAF Constrain			
ID	L2	PVB	#Shots	TAT	L2	PVB	#Shots	TAT	L2	PVB	#Shots	TAT
Case 1	41,597	48,402	400	1.82	42,754	49,727	361	1.86	37,872	26,110	1934	1.50
Case 2	37,053	42,959	419	1.75	38,001	45,878	351	1.85	28,850	36,490	1643	1.48
Case 3	75,930	77,676	541	1.81	76,147	80,372	496	1.82	65,601	75,100	1649	1.50
Case 4	17,626	31,198	301	1.80	19,378	33,850	287	1.80	12,542	25,319	1452	1.51
Case 5	31,831	52,884	525	1.86	33,062	54,024	490	1.78	27,668	49,799	1843	1.49
Case 6	33,639	49,523	418	1.80	34,998	51,136	384	1.77	28,918	44,730	1975	1.52
Case 7	25,336	42,035	413	1.77	27,569	43,503	388	1.75	10,646	37,494	1610	1.54
Case 8	14,674	27,317	382	1.79	15,334	29,381	355	1.75	13,697	21,310	1465	1.46
Case 9	38,837	58,092	609	1.86	39,926	56,459	562	1.73	35,237	55,033	1839	1.56
Case 10	12,917	23,046	303	1.78	14,928	24,707	271	1.72	9295	18,266	1057	1.44
Average	32,444	45,313	431	1.80	34,210	46,903	395	1.78	**27,033**	**38,965**	**1647**	**1.50**
Ratio	1.20	1.16	0.26	1.20	1.27	1.20	0.24	1.19	**1**	**1**	**1**	**1**

## Data Availability

All data are included in the study.
